# Expanding health coverage in India: role of microfinance-based self-help groups

**DOI:** 10.1080/16549716.2017.1321272

**Published:** 2017-05-31

**Authors:** Somen Saha

**Affiliations:** ^a^ Indian Institute of Public Health, Gandhinagar, India​

**Keywords:** Microfinance, self-help group, health access, India

## Abstract

To fulfil its commitment to universal health coverage, it will be necessary for the Indian government to expand access to appropriate and affordable health services. Through the mechanism of microfinance-based self-help groups (SHGs), poor women and their families are provided not only with access to finance to improve their livelihoods but also, in many cases, with a range of basic health services. Governments and non-governmental organisations in India have implemented large-scale programmes for the promotion of SHGs. With 93 million people organised nationally, the SHGs provide an established population base that can potentially be used to extend health coverage. However, the potential for working with SHGs to improve people’s access to health services has not been an active part of the national policy discourse.​

​To fulfil its commitment to universal health coverage (UHC), it will be necessary for the Indian government to expand access to appropriate and affordable health services. Through the mechanism of microfinance-based self-help groups (SHGs), poor women and their families are provided not only with access to finance to improve their livelihoods but also, in many cases, with a range of basic health services. Microfinancing institutions have been in existence on the Indian subcontinent for centuries [1,2]. In modern India, the growth of the microfinance movement was preceded by a need to improve financial access for poor, the overwhelming majority of whom were concentrated in rural areas and dependent on money lenders [3]. Governments and non-governmental organisations in India have implemented large-scale programmes for the promotion of SHGs. With 93 million people organised nationally, the SHGs provide an established population base that can potentially be used to extend health coverage. While microfinance-based SHGs have essentially been a tool for poverty alleviation, the nature of the microfinance transaction – where women get together in SHGs on a regular basis to repay loans and deposit savings – promotes group solidarity, trust, and mutual support. Microﬁnance can improve long-term development, as women are the main brokers of children’s health and education [4]. Globally, there is emerging evidence to show that microfinance programmes have created non-financial benefits, including improved health, hygiene, and sanitation. However, the potential for working with SHGs to improve people’s access to health services has not been an active part of the national policy discourse.

The presence of an SHG centred around microfinance activities was associated with improvements in key aspects of health knowledge and in some behaviours. An analysis of nationally representative survey data found that the presence of an SHG was associated with significantly higher odds of women delivering their babies in an institution, feeding colostrum to the newborn, having knowledge of modern family planning methods, and using family planning products and services [5].

Furthermore, a field study found that the inclusion of a health programme within microfinance-based SHGs, such as health awareness sessions during routine SHG meetings, followed by home visits from village health workers, was associated with improvements in health behaviours, including facility-based deliveries, feeding newborns colostrum, and having a toilet at home [6]. While the health behaviours measured in this study were not comprehensive, the recorded improvements in sanitation, facility-based deliveries, and immediate breastfeeding indicate an increase in health-promoting behaviours among SHGs with a health programme. Although some health behaviours improved in the SHG study areas, others did not. In particular, there was no significant reduction in the outcome variable of diarrhoea among children and the programme had no effect in reducing household money spent on healthcare. This mixed result is not uncommon among research on this subject. Possible reasons for this mixed programme effect in the study areas are the inexperience of health workers, initial challenges in programme delivery, shortcomings in motivating the community, and traditional community beliefs about health issues.

With the scale and reach of the existing SHG programmes, findings from the above studies indicate that there is potential for these groups to be effectively harnessed to reach more women and their families with programmes that promote desired health behaviours, which, in turn, are expected to improve health outcomes.

It is probable that such outcomes are not limited to the context of India but may have a wider international significance. Studies of similar situations in both Bangladesh and Indonesia also demonstrate that the introduction of microfinance facilities can help to improve health outcomes. In Indonesia, an analysis of the Indonesian Family Life Survey data found that the presence of microfinance-based SHGs was associated with a positive change in children’s health [7]. In Bangladesh, an analysis of a household-level panel data set collected over 8 years found that access to microcredit enabled households to insure against health shocks [8].

To further generalise these findings for wider application, a key issue is to identify the pathways through which this association operates. If the pathways are understood, further interventions can be designed to strengthen both the administrative mechanism – the SHG – and the health programme, and this could contribute to improved health outcomes across India more broadly. A review of the published literature indicates several different pathways through which the presence of a microfinance organisation facilitates healthcare gains [1–9]. These include: (i) participation in a microfinance programme improves the socio-economic situation of the members by reducing some of the ill effects of income inequity through consumption smoothing; (ii) participation in a microfinance programme offers non-financial benefits, including improved health, hygiene, and sanitation, and the longer the duration of association the better the health outcome; (iii) microfinance with an associated programme to raise health awareness is linked to improved access to antenatal care, safe delivery and immunisation, and reductions in infant mortality and birth rate; (iv) financing healthcare costs through microinsurance and health loans has been linked to increased utilisation of healthcare services, and better financial protection against the costs of care; and (v) affordable healthcare products and services, such as non-clinical family planning methods, and point-of-use water treatment products, provided through SHGs have been linked to the greater awareness and increased adoption of such products. When members are associated with an SHG programme for a longer duration they are more likely to adopt better health behaviours and practices. Taken together, there is a strong indication that SHGs promote healthy behaviours. In addition, the healthcare gains are even greater when there is a health programme compared to when there is not.

The factors that drive and keep families in poverty are multidimensional, yet often our solutions are not. Poor families struggle to access affordable services that meet their basic needs, including healthcare, finance, education, and housing. No one sector can provide an appropriate package of services to help the poor to escape poverty. The positive role of microfinance-based SHGs in improving maternal and child health indicators observed in the above studies suggests a mechanism for making improvements in health coverage and addressing the health needs of poor women and their families. There are indications that existing SHGs could be leveraged to make progress in improving the coverage of existing publicly funded financial protection and health insurance schemes. There are additional reasons, from a social perspective, for investigating the possible positive impact of these programmes, including the broad population coverage of SHGs and the social capital that they produce.

There has been interest among federal and state governments and non-governmental organisations to invest in the SHG movement for livelihood generation and financial inclusion among poor women and their families. There is a bigger role for the government and SHG federations in supporting individual SHGs as well as integrating health programmes within the broader SHG development and livelihood programmes.

Finally, public health planners could leverage SHGs to increase the proportion of the population enjoying health coverage and make progress to improve financial coverage and utilisation of existing publically financed health protection schemes, although a lot more work is needed to optimise these possibilities. With their broad population coverage, SHGs present an administrative apparatus to reach poor women and their families with essential health programmes. Public health planners should invest in further investigating the role of existing SHG programmes to expand health coverage among the difficult-to-reach population, particularly poor women and their families.

## Paper context

This paper explores the potential for existing microfinance networks, using self-help groups (SHGs) with attached health programmes, to contribute to improved health coverage for the poor. We found that SHGs present an administrative apparatus to reach poor women and their families with essential health programmes. Public health planners should invest in further investigating the role of existing SHG programmes to expand health coverage among the difficult-to-reach population, particularly poor women and their families.
